# An Improved TDOA Method for Land-Based Long-Range HF Skywave Source Geolocation and Experimental Results

**DOI:** 10.3390/s23010507

**Published:** 2023-01-02

**Authors:** Chen Xu, Hongtao Cai, Shunzu Gao, Qingwei Zhai

**Affiliations:** 1 School of Electronics and Information, Wuhan University, Wuhan 430072, China; 2 Hubei Luojia Laboratory, Wuhan 430072, China; 3 The 54th Research Institute of China Electronics Technology Group Corporation (CETC 54), Shijiazhuang 050000, China

**Keywords:** high frequency (HF) source geolocation, ionosphere, non-line-of-sight (NLOS), time-difference-of-arrival (TDOA)

## Abstract

The real-time information of the unknown ionospheric environments is difficult to obtain, plaguing the timely and accurate geolocation of high frequency (HF) sources. In this paper, we propose an improved HF skywave source geolocation method based on the time-difference-of-arrival (TDOA) with the semidefinite programming (SDP), and model HF signal propagation paths as paths with significant non-line-of-sight (NLOS) biases. With this method, no priori information about the ionosphere, especially the priori ionospheric virtual heights of reflection, is necessary while timely and accurately geolocating the HF sources. Furthermore, we use the ray tracing technique and build a 3D ionospheric electron density gridded matrix model to simulate realistic HF signal propagation paths. In the simulations, the proposed method is compared with existing methods, and detailed geolocation error distribution maps are given. In the experiments, HF I/Q data captured from different types of HF transmitters are located by six receivers with time synchronization. Simulated and experimental results show that the proposed method improves the positioning accuracy by about 50% compared with existing methods under the same conditions, and the average relative positioning error is less than 2.7%.

## 1. Introduction

High-frequency (HF) communications, which propagate over long distances by ionospheric reflections, are widely used in shortwave broadcasting, civil aviation, and military communication [1]. HF transmission has also become an ideal communication method for land or sea search and rescue due to its strong anti-interference ability, long communication distance, easy system maintenance, and low cost. The priori ionospheric information can reduce the time delay difference introduced by the ionosphere, thus further improving the geolocation accuracy of the HF source [2]. However, rapid access to large-scale real-time priori ionospheric information is daunting, leading to slower geolocation and more extensive search and rescue ranges. Therefore, timely and accurate geolocation of high-frequency sources in unknown ionospheric environments is a pressing problem [3].

In recent years, passive radio frequency (RF) measurements are often used to locate HF sources. Many geolocation methods have been proposed, such as received signal strength (RSS)-based geolocation [4], time-of-arrival (TOA)-based geolocation [5], time-difference-of-arrival (TDOA)-based geolocation [6], and angle-of-arrival (AOA)-based geolocation [7], as well as joint methods [8]. The traditional HF source geolocation method is based on the AOA system, also called the direction-finding (DF) system, using a large-scale antenna array to measure the AOA of the received signal and through triangulation to locate HF sources [7,9,10,11]. However, DF systems with large-scale antenna arrays have many drawbacks, such as being costly and environmentally unfriendly, being complicated to maintain, and occupying a large area. During the long-distance propagation of HF signals, even minor angle errors can lead to significant distance deviations. In addition, the continuous deterioration of the electromagnetic environment has led to the decline of the DF system performance [12].

**Figure 1:**
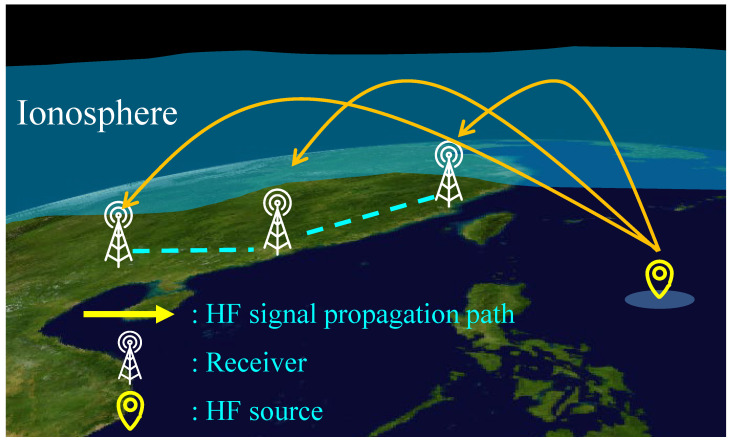
Schematic diagram of HF skywave source geolocation.

Hence, improved TDOA-based methods have been proposed to solve HF geolocation performance degradation. The TDOA technique is mature for positioning systems with unknown signal transmission time, but there is little research on TDOA-based HF skywave geolocation systems. A schematic diagram of TDOA-based HF skywave source geolocation is shown in Figure 1. The method in [12] proposed a nonlinear filtering algorithm for state estimation with symmetric alpha-stable noise presented, and the average geolocation error is less than 75 km. Multiple receivers capable of synchronizing HF radio signals are built in [13], and the HF source is estimated using the geolocation method based on the TDOA. These methods achieve high geolocation accuracy under favorable conditions in the experiments. However, these methods assume that the lower boundary of the ionosphere is at the same height everywhere [13], and require priori or estimated information about the ionospheric virtual reflection heights. Nevertheless, in practice, the ionosphere is irregular and varies in height. When real-time ionospheric path assessments are unavailable (or unreliable) due to a lack of proper data, the signal reflection heights and propagation paths in the ionosphere are virtually unknown [3]. It brings tremendous difficulties to the real-time and high-precision HF source TDOA-based geolocation. Thus, new methods for HF source geolocation are still worth discovering.

In this paper, an improved TDOA geolocation method based on semidefinite programming (SDP) is proposed for accurately locating HF skywave signal sources in real-time. Generally, HF skywave signal propagation paths are non-line-of-sight (NLOS), and the ground distances between the transmitter and the receiver are much larger than the reflection height of the ionosphere [12]. In previous publications, we proposed two-step localization methods based on TDOA for the unmanned aerial vehicle (UAV) [8]. However, it cannot be directly used in HF geolocation since the skywave propagation model is not considered. Therefore, we improved the method in [8] to make it applicable to HF source geolocation. We model HF signal propagation paths as paths with significant NLOS biases and build a new TDOA geolocation math model. Then, we use the ray tracing technique and introduce a real 3D ionospheric electron density model (explained in Appendix A) to simulate the HF signal propagation path for more realistic TDOA data. Finally, the proposed method uses TDOA data observed by receivers to optimize the HF source location. The proposed method does not require priori information about ionospheric virtual heights of reflection or other statistics. In simulations and experiments, the proposed method improves the geolocation accuracy and significantly reduces the search range of HF sources compared with other methods.

The remainder of this paper is organized as follows. The system model is described in Section 2. The HF geolocation method based on TDOA is described in Section 3. The simulations performance and experimental geolocation results are assessed in Section 4. Finally, Section 5 gives the conclusions.

## 2. System Model

**Figure 2:**
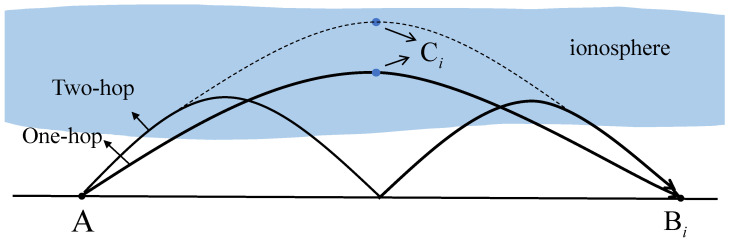
Schematic diagram of geometric relationship between the high-frequency (HF) source, the ith apogee and the ith receiver.

The schematic representation of the proposed HF sources geolocation scheme is shown in Figure 2. We consider the HF geolocation scenario that includes a stationary transmitter and multiple receivers in a geodetic coordinate system. Generally, the propagation path of HF signals is unknown due to the irregularity of the ionosphere. Researchers usually approximate it as a one-hop or multi-hop path model [13]. The monolayer quasi-parabolic (QP) model is a simple and effective model for calculating the radiation parameters considering an ionospheric medium, defined by the parabolic equation of the electron density versus height. The QP model has been a time-tested means of ionospheric radio propagation analysis [14], and it has been successfully utilized for HF source geolocation based on the sensor-collected TDOA observations for ionosphere-refracted radio rays [13,15,16].

Furthermore, using one-hop QP mode, communication is possible over distances in the range of 3000 km. In this paper, we consider one-hop paths to illustrate our method because the one-hop QP model [17,18] can achieve a good trade-off [19]. Note that it does not mean our method cannot be applied to multi-hop paths or models.

Consider a TDOA system with *M* time-synchronized receivers, where the receiver that first receives the HF signal is used as a reference without loss of generality, with subscript 1 to represent the reference receiver. The HF source is located at $A={\left[\chi ,\psi \right]}^{T}$, where χ presents the latitude and ψ presents the longitude. The ith receiver is located at $B_{i}={\left[\chi_{bi},\psi_{bi}\right]}^{T},i=1,2,\ldots ,M$. To facilitate the following calculation, we convert the latitude and longitude coordinate system into World Geodetic System 1984. Thus, $A={\left[x,y,z\right]}^{T}$ and $B_{i}={\left[x_{bi},y_{bi},z_{bi}\right]}^{T}$ denote the locations of the HF source and the ith receiver. $C_{i},i=1,2,\ldots ,M$ denotes the apogee of the ith HF signal propagation path without actual use. As shown in Figure 2,

(1) $$r_{i1}=\left({\overset{⏜}{AC}}_{i}+\overset{⏜}{C_{i}B_{i}}\right)-\left({\overset{⏜}{AC}}_{i}+\overset{⏜}{C_{1}B_{1}}\right)+n_{i1},i=2,3,\ldots ,M,$$

where $r_{i1}$ denotes the distance difference between the HF signal propagation from the HF source to ith and the reference receiver, measured by TDOA. ${\overset{⏜}{AC}}_{i}$ and $\overset{⏜}{C_{i}B_{i}}$ denote the real HF signal propagation distances from the HF source to the ith apogee and from the ith apogee to the ith receiver, respectively; $n_{i1}=n_{i}-n_{1}$, $n_{i}$ is the range measurement noise for the receivers, which can be modeled as a zero-mean white Gaussian random variable with a variance $\sigma_{i}^{2}$. As mentioned in Section 1, $\overset{⏜}{{\mathrm{AC}}_{i}B_{i}}$ is much greater than the perpendicular distance from point $C_{i}$ to the earth surface. Combined with the NLOS signal propagation idea, we rewrite Equation (1) as

(2) $$r_{i1}=\left(d_{i}-d_{1}\right)+e_{i1}+n_{i1}=\Delta t_{i1}c,i=2,3,\ldots ,M,$$

where $d_{i}={\overset{⏜}{AB}}_{i}={∥A-B_{i}∥}_{2}$ denotes the true distances (the straight-line distances) from the HF source to the ith receiver (${∥\cdot ∥}_{2}$ denotes the 2-norm); $e_{i1}=e_{i}-e_{1}$, $e_{i}={\overset{⏜}{AC}}_{i}+\overset{⏜}{C_{i}B_{i}}-{\overset{⏜}{AB}}_{i}$ represents the NLOS-caused positive bias, $e_{i}$ is much smaller than $d_{i}$; *c* is the speed of light; $\Delta t_{i1}$ is the time difference of arrival, which can be expressed as $\Delta t_{i1}=t_{i}-t_{1}$, with $t_{i}$ and $t_{1}$ being the time instant when the signal arrives at the ith and reference receiver, respectively. Since the receiver that receives the HF signal first is used as a reference, thus

(3) $$t_{i}\geq t_{1},i=2,3,\ldots ,M.$$

## 3. Geolocation Method

In this section, we present the detailed recipe of the proposed geolocation method. From Equation (2), we can easily obtain

(4) $$n_{i1}=\Delta t_{i1}c-\left(d_{i}-d_{1}\right)-e_{i1},i=2,3,\ldots ,M.$$

According to the geometrical relationship between the locations of the ith receiver and HF source, together with the HF signal propagation path, we have

(5) $$\begin{array}{c} {\overset{⏜}{AC}}_{i}+\overset{⏜}{C_{i}B_{i}}>{\overset{⏜}{AB}}_{i}\\ \Rightarrow {∥A-C_{i}∥}_{2}+{∥C_{i}-B_{i}∥}_{2}>{∥A-B_{i}∥}_{2}. \end{array}$$

Based on the geometrical relationship between the ith and jth receiver locations, we have

(6) $$\begin{array}{c} {\overset{⏜}{AB}}_{i}+{\overset{⏜}{AB}}_{j}>\overset{⏜}{B_{i}B_{j}}\\ \Rightarrow {∥A-B_{i}∥}_{2}+{∥A-B_{j}∥}_{2}>{∥B_{i}-B_{j}∥}_{2},i\neq j. \end{array}$$

Hence, the geolocation model, which tries to minimize the sum errors of distance estimation at *M* receivers, can be defined as

(7) $$\begin{array}{c} \underset{d_{i},d_{1},e_{i1}}{\mathrm{minimize}}\;\sum_{i=1}^{M}(\Delta t_{i1}c-\left(d_{i}-d_{1}\right)-e_{i1}),\\ \mathrm{subject}\;\mathrm{to}\;\;\mathrm{Equations}\;(3),\;\left(5\right)\;\mathrm{and}\;\left(6\right). \end{array}$$

To make Equation (7) obtain the optimal global solution, it must be secondarily differentiable. Doing some algebraic operations on Equation (4), then squaring the two sides and moving the noise term to the right side, we have

(8) $$\begin{array}{c} \Delta t_{i1}^{2}c^{2}-d_{i}^{2}-d_{1}^{2}+2d_{i}d_{1}-e_{i1}^{2}-2e_{i1}\left(d_{i}-d_{1}\right)=2n_{i}\left(d_{i}-d_{1}+e_{i1}\right)+n_{i}^{2}\\ =\varepsilon_{i}. \end{array}$$

Let

(9) $$q_{i}=e_{i1}^{2}+2e_{i1}\left(d_{i}-d_{1}\right),$$

(10) $$v_{i}=2d_{i}d_{1}.$$

Since the measurement noise $n_{i}^{2}$ is usually much smaller than $\left|d_{i}-d_{1}+e_{i1}\right|$ in practice, $\left|\cdot \right|$ denotes absolute value, and the $n_{i}^{2}$ term in Equation (8) can be ignored. Equation (8) is simplified to

(11) $$\varepsilon_{i}=\Delta t_{i1}^{2}c^{2}-d_{i}^{2}-d_{1}^{2}+v_{i}-q_{i}.$$

Therefore, the nonlinear least-squares estimator with unknown parameters *d*, *v*, *q* can be expressed as

(12) $$\begin{array}{c} \underset{d_{i},d_{1},v_{i},q_{i}}{\mathrm{minimize}}\;\sum_{i=1}^{M}w_{i}(\Delta t_{i1}^{2}c^{2}-d_{i}^{2}-d_{1}^{2}+v_{i}-q_{i}{)}^{2},\\ \mathrm{subject}\;\mathrm{to}\;\;\mathrm{Equations}\;(3),\;(5),\;(6),\;\left(9\right)\;\mathrm{and}\;\left(10\right), \end{array}$$

where $w_{i}$ is a positive weight. The main function of the weights is to give the estimator the relative importance of each data. In general, the weights $w_{i}≃{\left(r_{i}^{2}\sigma_{i}^{2}\right)}^{-1}$ [20], where $r_{i}$ denotes the signal propagation distance. The cost function of the minimization problem in Equation (12) is still nonlinear and non-convex. Thus, the auxiliary variables are introduced as

(13) $$h_{i}=d_{i}^{2}={∥A-B_{i}∥}_{2}^{2},$$

(14) $$h_{1}=d_{1}^{2}={∥A-B_{1}∥}_{2}^{2}.$$

Equations (13) and (14) can be written in a matrix form by using the Schur complement [20]

(15) $$\begin{array}{c} h_{i}={\left[\begin{array}{c} B_{i}\\ -1 \end{array}\right]}^{T}\left[\begin{array}{cc} I_{3} & A\\ A & o \end{array}\right]\left[\begin{array}{c} B_{i}\\ -1 \end{array}\right]\\ \;\;\;\;={\left[\begin{array}{c} x_{bi}\\ y_{bi}\\ z_{bi}\\ -1 \end{array}\right]}^{T}\left[\begin{array}{cccc} 1 & 0 & 0 & x\\ 0 & 1 & 0 & y\\ 0 & 0 & 1 & z\\ x & y & z & o \end{array}\right]\left[\begin{array}{c} x_{bi}\\ y_{bi}\\ z_{bi}\\ -1 \end{array}\right], \end{array}$$

(16) $$\begin{array}{c} h_{1}={\left[\begin{array}{c} B_{1}\\ -1 \end{array}\right]}^{T}\left[\begin{array}{cc} I_{3} & A\\ A & o \end{array}\right]\left[\begin{array}{c} B_{1}\\ -1 \end{array}\right]\\ \;\;\;\;={\left[\begin{array}{c} x_{b1}\\ y_{b1}\\ z_{b1}\\ -1 \end{array}\right]}^{T}\left[\begin{array}{cccc} 1 & 0 & 0 & x\\ 0 & 1 & 0 & y\\ 0 & 0 & 1 & z\\ x & y & z & o \end{array}\right]\left[\begin{array}{c} x_{b1}\\ y_{b1}\\ z_{b1}\\ -1 \end{array}\right], \end{array}$$

where

(17) $$\left[\begin{array}{cc} I_{3} & A\\ A & o \end{array}\right]≽0.$$

*o* is a new variable and $I_{3}$ is 3 identity matrix. $d_{i}$ and $d_{1}$ are positive, $v_{i}$ in Equation (12) satisfies

(18) $$v_{i}\geq 0.$$

Since $q_{i}$ is an unstable model variable, a constraint is imposed on $q_{i}$ according to the physical propagation model to improve its estimation accuracy. First, since the method is based on land-based long-range HF skywave geolocation, the HF sources are mostly outside the receiver surround range. Therefore, the HF signals propagate over long distances and in essentially the same propagation direction. Second, using the QP model, the properties of the ionosphere traveled by HF signals with approximately the same propagation direction are the same. Therefore, we assume that the receiver which first receives the HF signal (reference receiver) has the shortest HF signal propagation distance, which indicates that the HF source is the closest to the reference receiver [21]. Combined with the QP model, we have

(19) $$q_{i}=e_{i1}^{2}+2e_{i1}\left(d_{i}-d_{1}\right)>0.$$

Using the relaxation in Equations (15) and (16) and adding the constraint with Equations (18) and (19), the problem in Equation (12) can be expressed as [22]

(20) $$\begin{array}{c} \underset{h_{i},q_{i},v_{i}}{\mathrm{minimize}}\;\sum_{i=1}^{M}w_{i}(\Delta t_{i1}^{2}c^{2}-h_{i}-h_{1}+v_{i}-q_{i}{)}^{2}\\ \;\;\;\;\;\;\;\;\;\;\;\;\;\;\;\;+\;\delta \sum_{i=1}^{M}q_{i}^{2}\;+\;\gamma \sum_{i=1}^{M}v_{i}^{2},\\ \mathrm{subject}\;\mathrm{to}\;\;\;\;\mathrm{Eqsuations}\;(3),\;(5),\;(6),\;(15),\;(16),\;(17),\;\left(18\right)\;\mathrm{and}\;\left(19\right). \end{array}$$

The above equation is a typical SDP optimization problem and can be solved effectively with interior point methods [22]. Note that δ is a penalization factor which is required when the problem is ill-posed [20]. The γ makes the constraint in Equation (19) tend to choose an appropriate value of $v_{i}$ to ensure the feasibility of the constraint. The value of $v_{i}$ in Equation (20) is optimized to make the constraint tighter.

This proposed method resolves the problem of low accuracy in geolocating HF sources in unknown ionospheric environments. Additionally, with this method, no priori information about the ionosphere, especially the priori ionospheric virtual heights of reflection, is necessary while timely and accurately geolocating the HF sources.

## 4. Simulation and Experimental Results

### 4.1. Simulation Setup and Results

In this section, simulations are performed to validate the effectiveness of the proposed method. The HF source and receivers are randomly distributed in the region with latitudes ranging from $10^{\circ }\;N$ to $50^{\circ }\;N$ and longitudes ranging from $90^{\circ }\;E$ to $160^{\circ }\;E$.

We use the ray tracing method and introduce a realistic ionospheric 3D electron density gridded matrix model to simulate HF signal propagation paths. A detailed explanation of the 3D ionospheric model is given in Appendix A. The 3D gridded matrix model of the ionosphere electron density is at altitudes ranging from 0 to 1000 km. The gridded matrix elements properties of the ionospheric propagation medium are specified using the Internation Reference Ionosphere (IRI) model with the grid accuracy of $1^{\circ }\times 1^{\circ }\times 1\;\mathrm{km}$ (Latitude×Longitude×Height). The applicability and limitations of the IRI model are discussed in Appendix A. We use the methods in [23] to generate the received signals. For each link, the optimal weights should be set according to its NLOS bias. Since the HF signal propagation paths are unknown in practice, the weight elements $w_{i}$ are all set to 1 to reduce the complexity of the problem [8]. Previous studies show such an approximation does not degrade the performance significantly [24]. The penalization factor is set as δ=0.1, and γ is set as 1 [25]. In addition, the proposed method is implemented by the CVX toolbox in MATLAB [26,27]. All the simulations are carried out on a computer with 32 GB memory and a 5.0 GHz CPU (Intel Core i7-12700K). The proposed method is compared with the existing geolocation methods: (1) The method proposed in [12] which maximizes the crosscorrelation function to estimate TDOAs in the first step and derives the target location based on Kalman particle filtering, denoted by KPF. (2) The method proposed in [13] is based on TDOA Quasi-Parabolic mode, denoted by TQP.

To test the performance of the proposed method, simulations with 100 independent trials are conducted. We calculate the mean square error (MSE) of the estimated position with the Euclidean distance as follows:

(21) $$\mathrm{MSE}=\frac{1}{100}\sum_{k=1}^{100}{∥\alpha -\hat{\alpha }\left(k\right)∥}_{2},$$

where α denotes the real position of the HF source, and $\hat{\alpha }\left(k\right)$ is the estimated position of the HF source at the $k^{th}$ independent trial. Moreover, the Cramer–Rao lower bound (CRLB) for TDOA systems in NLOS conditions [28] is used as the performance benchmark.

**Figure 3:**
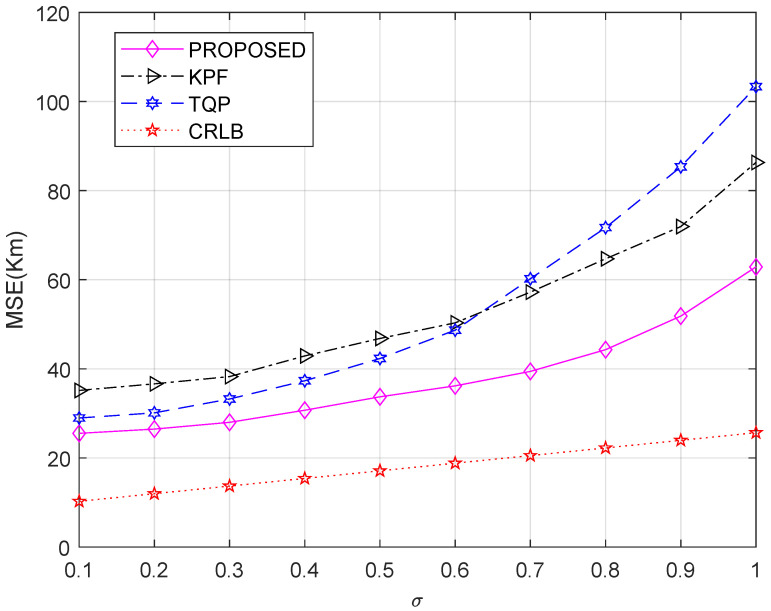
MSE of HF source position estimation with different values of σ.

**Figure 4:**
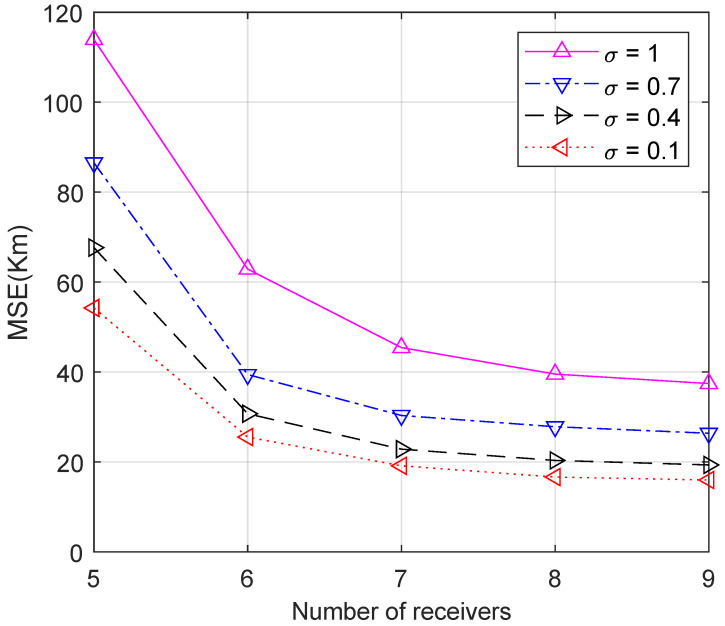
MSE of HF source position estimation with different numbers of receivers and different values of σ.

First, we test the performance of the proposed method and compare it with other methods. The HF signal frequency is set to 7.23 MHz. The time of the IRI model is set at 9:00 UT on 13 May 2020. Figure 3 shows the performance comparison with 100 independent geolocation trials of the proposed method and other methods using six receivers in different σ conditions. As σ increases from 0.1 to 0.5, the proposed method is slightly better than the others. When σ>0.6, indicating a large measurement noise, the MSEs and interference immunity of the proposed method are significantly better than the other methods. Figure 4 shows the performance with different numbers of receivers and different σ. Limited by the number of unknown variables, the proposed method cannot achieve better results when the number of receivers is less than 6 (M<6). When M≥6, the error decreases and tends to the limit as the number of receivers increases. Figure 3 and Figure 4 illustrate that the proposed method outperforms existing algorithms in terms of geolocation accuracy under the same conditions and can significantly suppress the effect of large noise.

**Figure 5:**
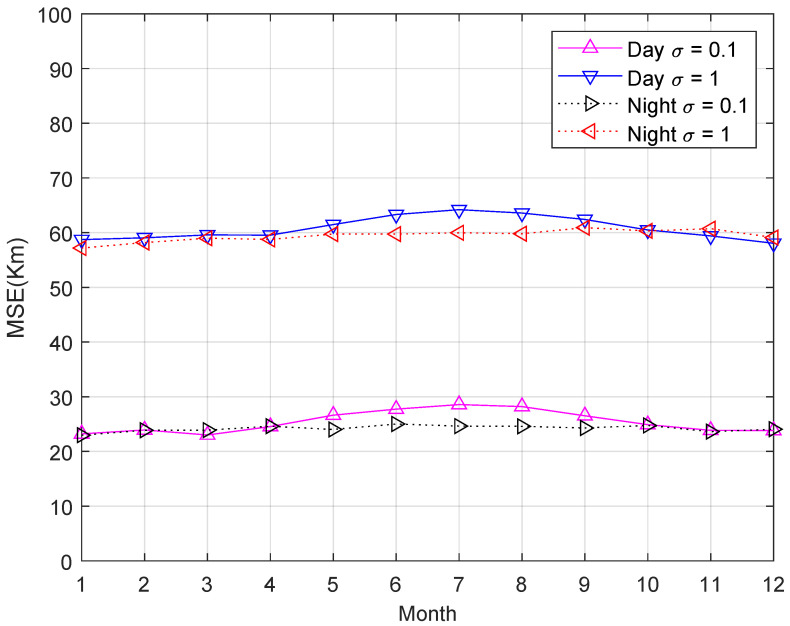
MSE of HF source position estimation in the daytime and nighttime in different seasons.

Next, we test the performance of the proposed method in the daytime and nighttime in different seasons. The IRI model is set to the 1st of every month, 03:00 UT for the day, and 15:00 UT for the night. Figure 5 shows the MSE of HF source position estimation in the daytime and nighttime in different seasons. From Figure 5, the MSEs are slightly higher in summer than in winter and slightly higher in the day than at night due to the influence of ionospheric properties. Unfortunately, due to the IRI model’s limitations, it is impossible to simulate the perturbed ionosphere or ionospheric irregularities. However, the proposed algorithm has good robustness in the simulation.

The station arrangement of receivers also significantly affects the accuracy of HF source geolocation. We test two different sets of receiver deployment locations to simulate the real scenario. The first set of six fixed receivers is located in Shenzhen, Beijing, Harbin, Kunming, Shanghai, and Wuyishan, and the time of the IRI model is set at 9:00 UT on 13 May 2020. The second set of six fixed receivers is located in Beijing, Chengdu, Shannxi, Shenzhen, Kunming, and Wuyishan, and the time of the IRI model is set at 15:00 UT on 8 September 2021.

**Figure 6:**
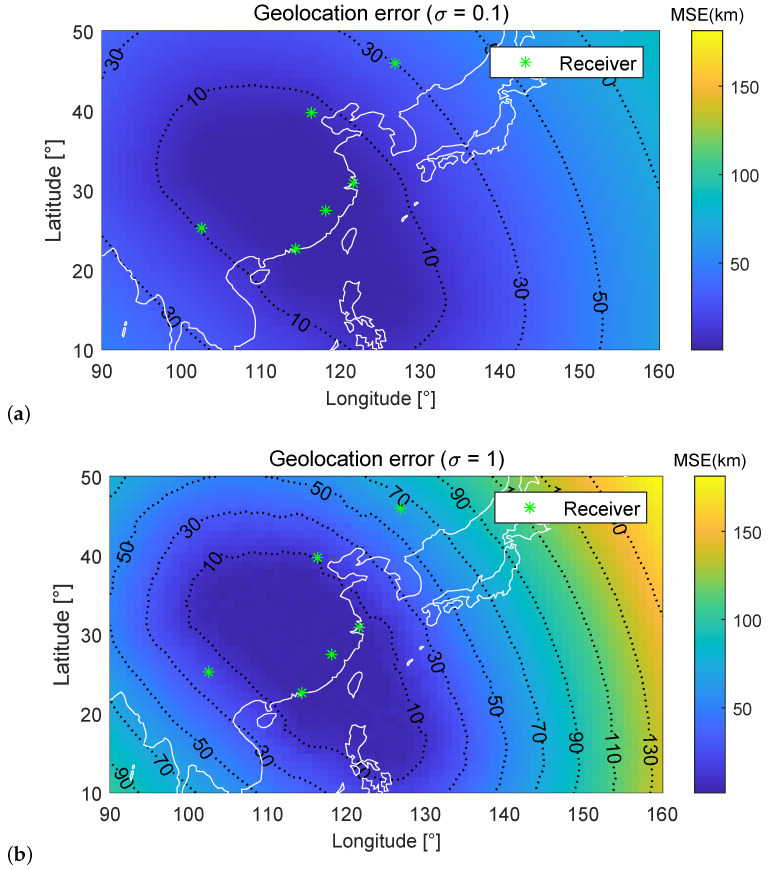
Geolocation error distributions for the first set of six fixed receivers under different σ conditions.

**Figure 7:**
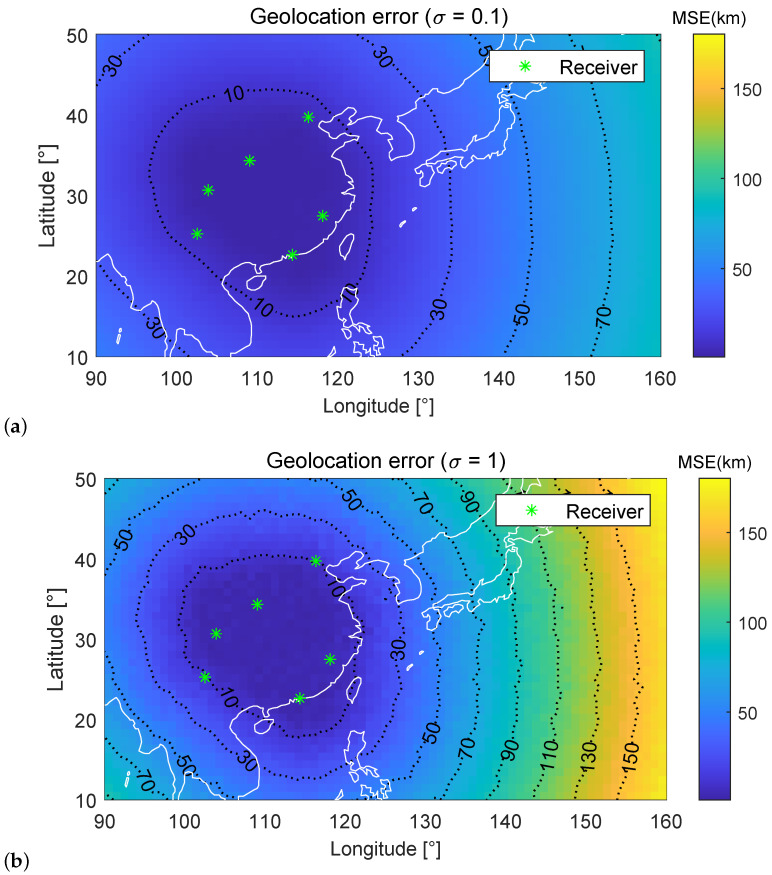
Geolocation error distributions for the second set of six fixed receivers under different σ conditions.

Figure 6 and Figure 7 show the geolocation error (in km) distribution of the first and second set of 6 fixed receivers under σ=0.1 and σ=1, respectively. Positions of six receivers are marked with asterisks. In Figure 6 and Figure 7, the simulation region is divided into grids with an accuracy of $1^{\circ }\times 1^{\circ }$ (Latitude×Longitude). The geolocation error of the HF source at each grid is tested individually, resulting in an error distribution of the entire region. Different receiver arrangement locations result in different geolocation error distributions. For example, when σ=0.1, the area with MSE < 10 km is 26.69% for the first set of six receivers, while the area with MSE < 10 km is 20.61% for the second set. However, geolocation error is generally proportional to the distance between the HF source and the receivers.

Table 1 and Table 2 give the percentage of the different MSE area under σ=0.1 and σ=1. In Figure 6 and Figure 7, subject to different levels of noise, Figure 6a and Figure 7a are smoother compared to Figure 6b and Figure 7b, respectively. It is noteworthy that the increase in noise has a minor impact on the blue region, also called the high-precision region, and a more significant impact on the orange region. When reducing the number of receivers, the high-precision region decreases, and the orange region increases sharply. This phenomenon is consistent with the optimization logic of the algorithm under the antenna deployment position. A good receiver deployment location can significantly improve the geolocation accuracy of HF sources in a specific area and minimize the search and rescue range. Table 3 gives the running time of the proposed method for a single localization with the different number of receivers.

### 4.2. Experimental Setup and Results

To further demonstrate the effectiveness of our algorithm in real-world scenarios, we use six shortwave signal receivers with GPS time synchronization to build a HF geolocation test system. In the experiment, the receivers capture the target signal synchronously through baseband I/Q sampling. Then, the I/Q data are processed for time difference estimation. The receivers use the Global Positioning System (GPS) and BeiDou Navigation Satellite System (BDS) timing clock to achieve strict synchronization of receivers time. The accuracy of the civilian GPS/BDS timing system is about 50–100 ns, which translates to a distance of about 15–30 m and does not affect the positioning algorithm [29].

**Figure 8:**
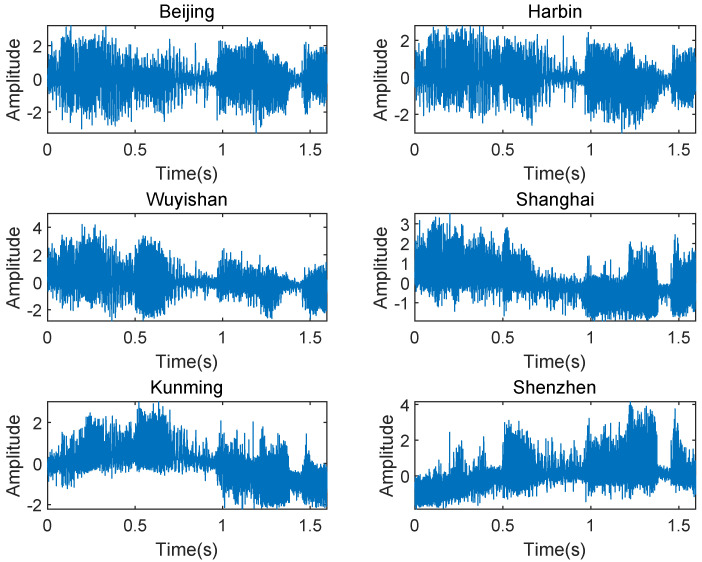
Received AM signals with the center frequency of 7.23 MHz.

We first verify the capability of locating an AM broadcast signal as shown in Figure 8. The HF source is located in Xi’an ($108^{\circ }61^{\prime }\;E,\;34^{\circ }37^{\prime }\;N$). The six fixed receivers are located in Beijing, Harbin, Wuyishan, Shanghai, Kunming, and Shenzhen. The received amplitude modulation (AM) signals captured at 9:15 UTC on 13 May 2020 have a center frequency of 7.23 MHz, a bandwidth of 5 kHz, a sampling rate of 10,240 Hz, and a duration of 1.6 s. The receiver located in Beijing is used as a reference. Table 4 shows the time delay estimation (TDE) obtained by the AM I/Q data of each receiver.

**Figure 9:**
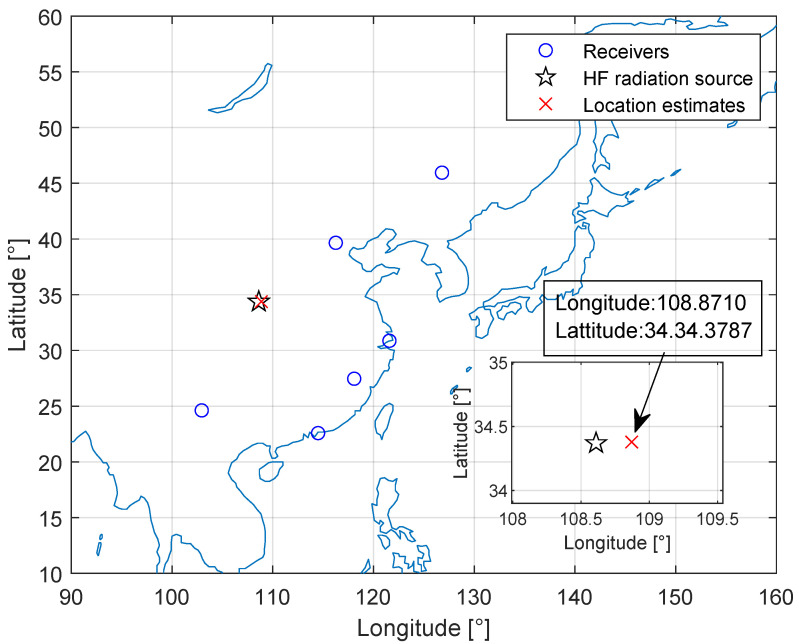
Experimental results of Xi’an HF source geolocation estimation.

Figure 9 shows the experimental result of Xi’an HF source geolocation estimation. The geographic locations of the receivers (marked with blue circles), the HF source (marked with a black star), and the estimated HF source (marked with a red cross) are shown in Figure 9. The geolocation error of the HF source is 23.97 km using the proposed method and the relative error is about 0.76%, calculated from dividing the absolute error by the maximum ground range from Harbin to Kunming [12], which is 3156.34 km.

**Figure 10:**
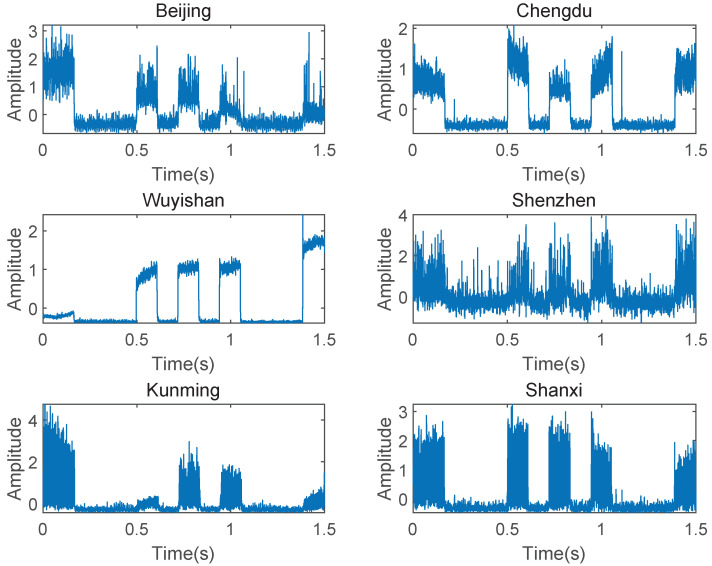
Received FSK signals with the center frequency of 8.433 MHz.

Then, we focus on a frequency shift keying (FSK) signal captured at 15:01 UTC on 8 September 2021 with the center frequency of 8.433 MHz over a 1.5 kHz band, a sampling rate of 8152 Hz, and a duration of 1.5 s, as shown in Figure 10. The FSK modulated signal is used by a coast radio station, which is located in Shanghai ($121^{\circ }54^{\prime }\;E,\;31^{\circ }11^{\prime }\;N$). The six fixed receivers are located in Beijing, Chengdu, Shannxi, Shenzhen, Kunming, and Wuyishan. The receiver located in Wuyishan is used as a reference. Table 5 shows the TDE obtained by the FSK I/Q data of each receiver.

**Figure 11:**
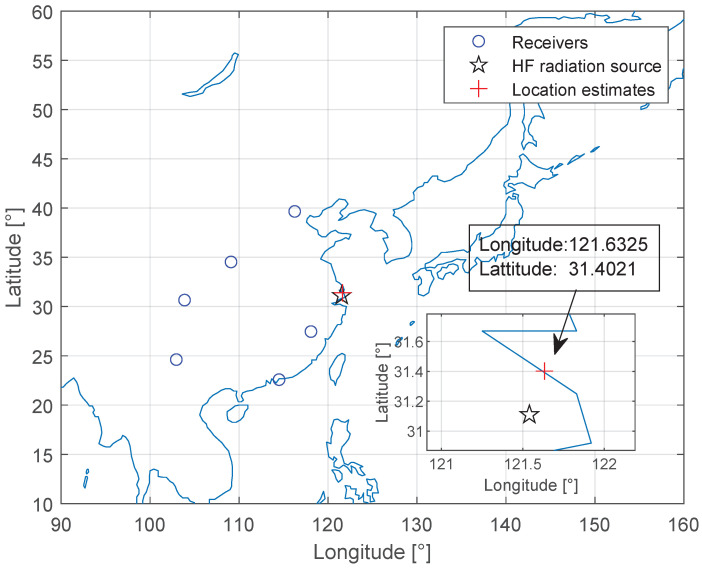
Experimental results of Shanghai HF source geolocation estimation.

The estimated Shanghai HF source (marked with a red plus marker) is shown in Figure 11. The geolocation error of the HF source is 33.65 km using the proposed method, and the relative error is about 1.61%, calculated from dividing the absolute error by the maximum ground range from Beijing to Kunming, which is 2084.56 km.

Finally, we assess all the sampled data in the time period in which AM and FSK are located, with a duration of 1.5 s for each set of data and an interval of 8.5 s between each measurement. As shown in Table 6, we summarize the maximum error and minimum error of the three geolocation methods. The average relative error of the proposed method is less than 2.7%, which improves the geolocation performance by about 50% compared with other methods. Under the same conditions, the proposed method has better noise immunity and the geolocation capability than other methods.

## 5. Conclusions

In this paper, we propose an improved TDOA geolocation method based on SDP, which models HF signal propagation paths as paths with significant NLOS biases. Then, the NLOS model is mathematized by the SDP method and solved by the interior point method. The proposed method does not require a priori ionospheric virtual height of reflection. Simulations and experiments verify the feasibility of the proposed method.

In the simulation, we use the ray tracing method and introduce realistic ionospheric 3D electron density gridded matrix model to simulate HF signal propagation paths. The geolocation performance of the entire region and detailed error distribution maps under different σ are given.

In the experiments, two sets of six time-synchronized receivers are deployed in different cities in China, and the HF I/Q signals are located algorithmically. The experimental results show that the average error of the proposed method is less than 2.7%, which is smaller than other existing methods. Moreover, the experimental estimation performance is quite close to the simulation estimation performance, which illustrates that the method is efficient for HF geolocation and significantly narrows the geolocation range. 

## Figures and Tables

**Table 1:** Percentage of areas with different MSEs using the first set of six fixed receivers deployment under σ=0.1 and σ=1.

	MSE < 10 km	MSE < 30 km	MSE < 50 km	MSE < 70 km	MSE < 90 km	Average MSE
σ=0.1	26.69%	62.07%	82.17%	96.53%	100.0%	28.07 km
σ=1	17.55%	34.35%	52.18%	66.44%	76.33%	59.92 km

**Table 2:** Percentage of areas with different MSEs using the second set of six fixed receivers deployment under σ=0.1 and σ=1.

	MSE < 10 km	MSE < 30 km	MSE < 50 km	MSE < 70 km	MSE < 90 km	Average MSE
σ=0.1	20.61%	55.54%	74.30%	88.77%	99.97%	33.76 km
σ=1	13.29%	28.65%	47.85%	61.73%	70.59%	70.90 km

**Table 3:** Running time of the proposed method under a different number of receivers.

	5 Receivers	6 Receivers	7 Receivers	8 Receivers	9 Receivers
Running time (s)	0.4841	0.6521	0.8829	1.1503	1.4001

**Table 4:** TDE results of AM I/Q data (km).

	Harbin	Wuyishan	Shanghai	Kunming	Shenzhen
TDOA (Convert to distance)	1280	311	461	513	560

**Table 5:** TDE results of FSK I/Q data (km).

	Beijing	Chengdu	Shenzhen	Kunming	Shanxi
TDOA (Convert to distance)	612	1209	638	1369	619

**Table 6:** Geolocation errors of different methods in experiments.

	Time	Geolocation Error Min., Error Max., Average Error (km)		
	(UTC)	PROPOSED	KPF	TQP
AM	9:00–9:40	23.97, 180.11, 42.65	65.49, 312.59, 87.02	51.33, 264.23, 83.16
FSK	14:50–15:30	33.65, 234.37, 56.32	74.27, 356.43, 126.88	80.58, 422.84, 127.74

**Figure A1:**
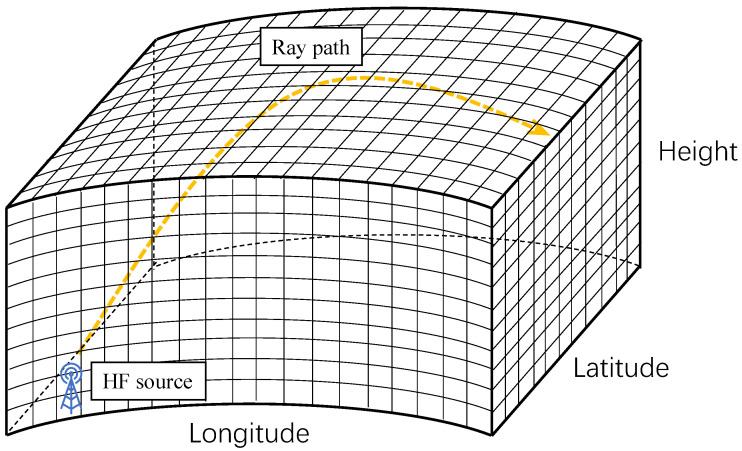
3D gridded matrix of the ionospheric electron density.

**Figure A2:**
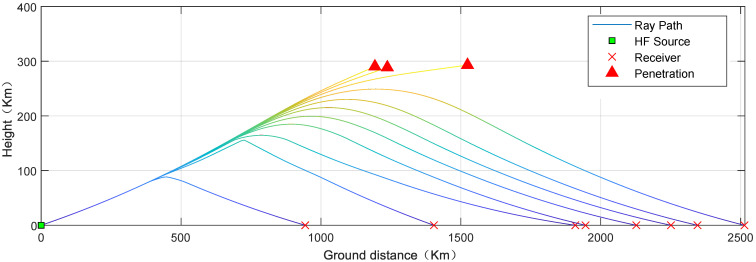
Simulation of HF signal ray tracing with different signal frequencies and a fixed emission angle of $10^{\circ }$.

**Figure A3:**
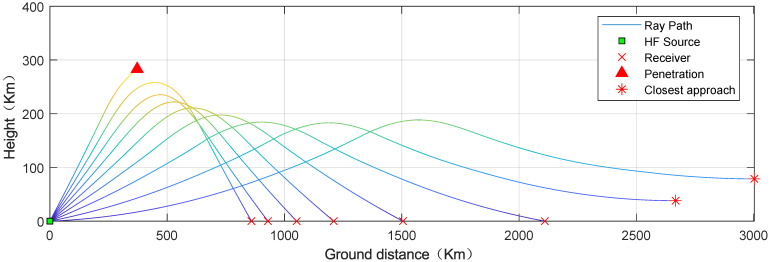
Simulation of HF signal ray tracing with different emission angles and a fixed signal frequency of 7.23 MHz.

**Figure A4:**
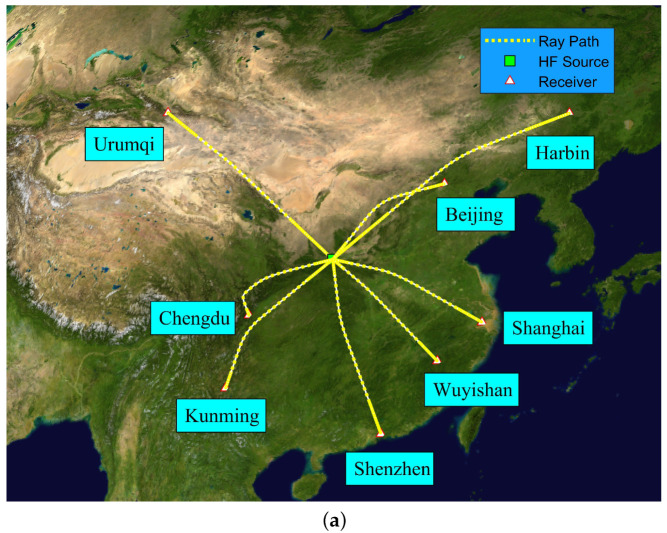

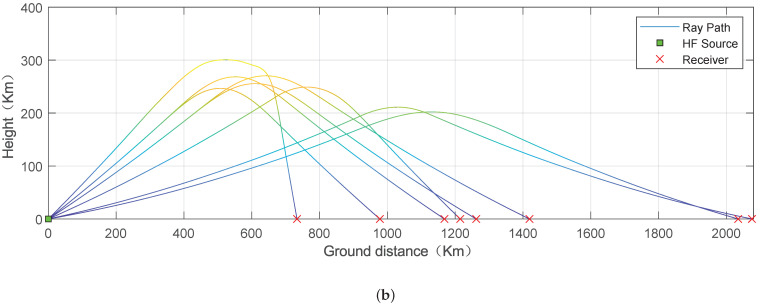
Simulating the HF signal path from Xi’an to eight receivers using ray tracing method. (**a**) 3D ray tracing path; (**b**) 2D ray tracing path.

## Data Availability

Not applicable.

## Appendix A. 3D Ionospheric Model

In order to make the HF signal propagation process close to reality, we use a 3D ionospheric model to build our simulation environment. The ray tracing technique is a method that approximates the energy of the electromagnetic field along with the ray propagation under certain conditions. HF propagation generally satisfies the geometrical optics approximation condition [30]. Therefore, the ray tracing technique can simulate the propagation path of HF signals in the ionosphere and realistically verify the effectiveness of the geolocation algorithm [31]. In order to better simulate HF signal propagation paths and obtain realistic TDOA data, we develop a gridded matrix model of ionospheric electron density. Figure A1 shows the schematic diagram of the gridded matrix model, where the yellow dashed arrow indicates the ray path from the HF source. Each gridded element represents the electron density of the ionosphere here. Its grid accuracy is Latitude×Longitude×Height, which can be adjusted as needed. Due to the long distances involved, the 3D gridded matrix has a spherical shell shape. The ray tracing algorithm integrates the following partial differential equations [32]:

(A1) $$\left\{\begin{array}{c} \begin{array}{c} \frac{dr}{d\tau }=\frac{\partial H}{\partial k_{r}},\\ \frac{d\theta }{d\tau }=\frac{1}{r}\frac{\partial H}{\partial k_{\theta }},\\ \frac{d\varphi }{d\tau }=\frac{1}{r\sin\theta }\frac{\partial H}{\partial k_{\varphi }},\\ \frac{dk_{r}}{d\tau }=-\frac{\partial H}{\partial r}+k_{\theta }\frac{d\theta }{d\tau }+k_{\varphi }\sin\theta \frac{d\varphi }{d\tau },\\ \frac{dk_{\theta }}{d\tau }=\frac{1}{r}\left(-\frac{\partial H}{\partial \theta }-k_{\theta }\frac{dr}{d\tau }+k_{\varphi }r\cos\theta \frac{d\varphi }{d\tau }\right),\\ \frac{dk_{\varphi }}{d\tau }=\frac{1}{r\sin\theta }\left(-\frac{\partial H}{\partial \varphi }-k_{\varphi }\frac{dr}{d\tau }+k_{\theta }r\cos\theta \frac{d\theta }{d\tau }\right),\\ \frac{dt}{d\tau }=-\frac{\partial H}{\partial \omega },\\ \frac{d\omega }{d\tau }=\frac{\partial H}{\partial t}, \end{array} \end{array}\right.$$

where *r*, θ, and φ are the coordinates of one point along the ray tracing path in spherical polar coordinate. $k_{r}$, $k_{\theta }$, and $k_{\varphi }$ are components of the wave vector. *H* is the Hamiltonian operator. ω is the angular frequency of the electromagnetic wave. τ is just a parameter in the equations that has no actual meaning. Then, the Hamiltonian operator can be written as

(A2) $$H=\frac{1}{2}\left[\frac{c^{2}}{\omega^{2}}\left(k_{r}^{2}+k_{\theta }^{2}+k_{\varphi }^{2}\right)-n^{2}\right],$$

(A3) $$n^{2}=1-X=1-\frac{f_{N}^{2}}{f^{2}}=1-\frac{N_{e}e^{2}}{4\pi^{2}f^{2}m_{e}\varepsilon_{0}},$$

where *n* is the phase refractive index, *c* is the speed of light, ω is the fixed angular frequency of the wave, *f* is the frequency of the electromagnetic wave, $f_{N}$ is the plasma frequency, $N_{e}$ is the electron density, *e* is the electron charge, $m_{e}$ is the electron mass, and $\varepsilon_{0}$ is the permittivity of vacuum. Then, the ray tracing path can be simulated by using the Runge–Kutta algorithm.

The gridded matrix elements properties of the ionospheric propagation medium are specified using the Internation Reference Ionosphere (IRI) model with the grid accuracy of $1^{\circ }\times 1^{\circ }\times 1\;\mathrm{km}$ (Latitude×Longitude×Height). The IRI model provides vertical profiles of electron density for one position, one date, and one time instant. The model is based on hourly-monthly medians. The IRI model has limitations because it is not developed to support ray propagation. However, large-scale 3D ionospheric electron density profile data are difficult to obtain, and IRI models can be an alternative. Furthermore, the ray-tracing-based simulation is closer to reality than the simple signal reflection simulation in the [12,13,21]. Note that the introduction of the ray tracing method only generates simulated data and does not have any effect on the geolocation method.

Figure A2 shows the simulation of the one-hop QP path at different frequencies with a fixed emission angle of $10^{\circ }$ using a 3D ionospheric model and ray tracing method. The time of the IRI model is set at 12:00 UT on 10 July 2020. The HF source is located at $109^{\circ }00^{\prime }\;E,\;34^{\circ }00^{\prime }\;N$ and emits HF signals to the south. The HF signal frequency is 1 MHz, 3 MHz, 5 MHz, 7 MHz, 9 MHz, 11 MHz, 13 MHz, 15 MHz, 17 MHz, 19 MHz, and 21 MHz, respectively. The red cross indicates the path landing point. The red triangle indicates that the path penetrates the ionosphere.

Figure A3 shows the simulation of the one-hop QP path with different emission angles and a fixed signal frequency of 7.23 MHz. The HF source is located at $109^{\circ }00^{\prime }\;E,\;34^{\circ }00^{\prime }\;N$ and emits HF signals to the south. The emission angle is $1^{\circ }$, $5^{\circ }$, $10^{\circ }$, $15^{\circ }$, $20^{\circ }$, $25^{\circ }$, $30^{\circ }$, $35^{\circ }$, and $40^{\circ }$, respectively. The red asterisk indicates that the signal is refracted through the ionosphere and failed to return to the ground.

As shown in Figure A4, we used a 3D ionospheric model to simulate the ray tracing path of HF signals transmitted from Xi’an. Eight receivers surround the HF source to represent different ionospheric environments located in Urumqi, Chengdu, Kunming, Shenzhen, Wuyishan, Shanghai, Beijing, and Harbin. The 3D gridded matrix model of the ionosphere electron density is at altitudes ranging from 0 to 1000 km. The HF signal frequency is 7.23 MHz. The time of the IRI model is set at 9:00 UT on 13 May 2020. The propagation of HF signals depends heavily on the ionospheric electron density. 3D ionospheric models combined with ray tracing methods make it more realistic to verify the geolocation method in simulations.
